# Increase in Reported Adverse Health Effects Related to Synthetic Cannabinoid Use — United States, January–May 2015

**Published:** 2015-06-12

**Authors:** Royal Law, Josh Schier, Colleen Martin, Arthur Chang, Amy Wolkin

**Affiliations:** 1 Division of Environmental Hazards and Health Effects, National Center for Environmental Health, CDC

On April 6, 2015, CDC received notification of an increase in telephone calls to U.S. poison centers related to synthetic cannabinoid use. Monthly calls to all poison centers are tracked by the National Poison Data System, which reported that adverse health effects or concerns about possible adverse health effects related to synthetic cannabinoid use increased 330% from 349 in January 2015 to 1,501 in April 2015. Synthetic cannabinoids include various psychoactive chemicals or a mixture of such chemicals that are sprayed onto plant material, which is then often smoked or ingested to achieve a “high.” These products are sold under a variety of names (e.g., synthetic marijuana, spice, K2, black mamba, and crazy clown) and can be sold in retail outlets as herbal products. Law enforcement agencies have regulated a number of these substances; however, manufacturers of synthetic cannabinoids frequently change the formulation to avoid detection and regulation. After the initial notification, CDC analyzed information from the National Poison Data System on reported adverse health effects related to synthetic cannabinoid use for the period January–May 2015.

During the 2015 study period, poison centers reported 3,572 calls related to synthetic cannabinoid use, a 229% increase from the 1,085 calls during the same January–May period in 2014 (Figure). The number of calls spiked notably in mid-April before decreasing nearly to 2014 levels by the end of May (Figure). Most calls concerned use among males (2,882 [80.7%]). Among 3,442 (96.4%) calls where age of the user was recorded, the median age was 26 years (range = 7 months–72 years).

The most commonly reported adverse health effects were agitation (1,262 [35.3%]), tachycardia (1,035 [29.0%]), drowsiness or lethargy (939 [26.3%]), vomiting (585 [16.4%]), and confusion (506, [4.2%]). Among 2,961 calls for which a medical outcome was reported, 335 (11.3%) callers had a major adverse effect (signs or symptoms that are life-threatening or result in substantial residual disability or disfigurement); 1,407 (47.5%) had a moderate effect (signs or symptoms that are not life-threatening and do not result in residual disability or disfigurement, but usually require some form of treatment). A total of 1,095 (37.0%) had a minor effect (signs or symptoms that are minimally bothersome and generally resolve rapidly with no residual disability or disfigurement), and 109 (3.7%) had no effect (1). A total of 15 (0.5%) deaths were reported.

Inhalation by smoking was the most common means of consumption (2,870 [80.3%]), followed by ingestion (698 [19.5%]). Most reported use was intentional (3,310 [92.7%]). Among 626 calls reporting use of synthetic cannabinoids with multiple substances, the most commonly reported other substances included alcohol (144 [23.0%]), plant-derived marijuana (103 [16.5%]), and benzodiazepines (69 [11.0%]). Only one of the deaths included reports of multiple substance use.

Calls indicating severe medical outcomes (major effect and death) were compared with calls indicating less severe outcomes (moderate effect, minor effect, and no effect). Results of a chi-square test demonstrated a significant association between sex and degree of severity. Males were significantly more likely to have a severe outcome (88.6%) than a less severe outcome (80.1%) (p<0.001). A significant association also was found between age group and severity (p<0.001); pairwise comparisons (adjusted by the stepdown Bonferroni procedure) indicated that persons aged 30–39 years and aged >40 years were significantly more likely than those aged 10–19 years to report a severe outcome (p = 0.001 and p<0.001, respectively).

The findings in this report are subject to at least two limitations. First, in some states, poison centers acted as central reporting centers for hospitals that evaluated persons experiencing a health effect associated with synthetic cannabinoid use. Situations in which a poison center was not contacted were not recorded, thus possibly underestimating the number of persons who were evaluated after synthetic cannabinoid use. Second, calls involving multiple substances were included in the analysis; therefore, adverse health effects might have resulted from use of other substances or a combination of substances.

The increasing number of synthetic cannabinoid variants available, higher toxicity of new variants, and the potentially increased use as indicated by calls to poison centers (2–3) might suggest that synthetic cannabinoids pose an emerging public health threat. Multiple other recent outbreaks (2–4) suggest a need for greater public health surveillance and awareness, targeted public health messaging, and enhanced efforts to remove these products from the market.

## Figures and Tables

**FIGURE:**
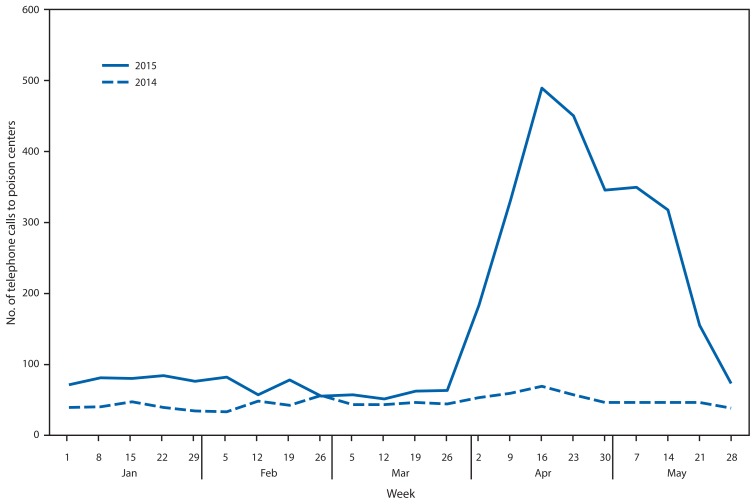
Number of telephone calls to poison centers reporting adverse health effects related to synthetic cannabinoid use, by week — National Poison Data System, United States, January–May 2014 and 2015
